# HTLV-1 Disease

**DOI:** 10.3390/pathogens10081001

**Published:** 2021-08-09

**Authors:** Chloé Journo, Fabiola Martin

**Affiliations:** 1 CIRI, Centre International de Recherche en Infectiologie, Retroviral Oncogenesis Team, Univ Lyon, Inserm, U1111, Université Claude Bernard Lyon 1, CNRS, UMR5308, ENS de Lyon, F-69007 Lyon, France; 2 School of Public Health, University of Queensland, Brisbane, QLD 4006, Australia; 3 Stonewall Medical Centre, Brisbane, QLD 4030, Australia

The years 2020 and 2021 will remain memorable years for many reasons. For *Pathogens* MDPI, it was the year where a special edition on Human T-cell Leukemia Virus Type 1 (HTLV-1) took centre stage, and for people living with HTLV-1 (PLHTLV), it was the year that the World Health Organisation adopted HTLV-1 as a health topic.

This sexually transmitted virus was identified in 1980 [1] but has been a human pathogen for thousands of years. However, the discovery of its extraordinarily high prevalence and associated morbidity and mortality amongst one of our culturally richest and oldest living Indigenous peoples in Central Australia shocked the scientific and general population world-wide in 2016 [2,3,4].

*Pathogen’s* Special Issue on HTLV-1 Disease aims to collate recent advances in basic science, clinical and epidemiological research enhancing our understanding of this virus’s pathogenicity. It is hard to believe, but it is true that 41 years since the discovery of HTLV-1, we still have no antiretroviral treatment that can cure this virus, prevent its transmission or avert disease from developing.

HTLV-1 infection is associated with the development of disease in approximately 5% of infected individuals. Although the vertical acquisition of HTLV-1 through breastfeeding has mostly been associated with the development of adult T-cell leukaemia/lymphoma (ATL), epidemiological data support the notion that vertical HTLV-1 transmission is also associated with HTLV-1-associated myelopathy (HAM). Strengthening this notion, Schwalb et al. provide data on the early-onset of HAM in Peru [5]. Investigation of familial HTLV-1 statuses indicates that most patients with early-onset HAM acquired HTLV-1 through breastfeeding. These data reinforce the need for public health enforced transmission prevention strategies.

The natural history of HTLV-1 infection requires further investigation focusing on different populations. Marcusso et al. report on a 20-year longitudinal cohort study of PLHTLV-1 in Sao Paulo, Brazil, and confirm that HAM disease is a significant predictor of early mortality [6]. In asymptomatic HTLV-1 carriers, HIV/HCV co-infection and advanced immunodeficiency are significant risk factors for death. Importantly, the mortality risk of people living with HTLV-1 and HIV is increased by several folds compared to people living with HIV only, suggesting synergy.

Two review articles also focus on the impacts of co-infections on the outcomes of HTLV-1 disease. Abelardo Araujo summarises eloquently the neurological sequelae of HTLV-1 or -2 with HIV-1 co-infection, where a potentially higher risk of HAM development or HAM-like myelopathy in co-infected individuals has been described [7]. However, Araujo also stresses the scarcity of recent and large-scale, longitudinal clinical studies to fully understand the pathogenesis of these co-infections. Dykie et al. summarise the current understanding of HTLV-1 and *Strongyloides stercoralis* co-infection [8]. *S. stercoralis* is an intestinal parasite highly prevalent in HTLV-1 endemic regions, known to cause hyperinfestation in PLHTLV. The review explains how HTLV-1 infection dysregulates the immune response increasing host susceptibility and increasing *S. stercoralis* pathogenicity. The strongyloides infestation seems to support higher HTLV-1 pro-viral load levels, maybe through an upregulation of oligoclonal proliferation, which is a risk factor for ATL development.

Marino-Merlo et al. provide an update on antiretroviral therapies tested in preclinical and clinical studies [9]. As a retrovirus, HTLV-1 might be sensitive to antiretrovirals that are used to target HIV-1. However, compared to HIV-1, the HTLV-1 replication cycle shows specificities that seem to limit the efficiency of HIV-1 drug repositioning. Most striking is the ability of HTLV-1 to replicate through clonal expansion of infected cells, in a reverse-transcriptase-independent process, in addition to the more classical infectious spread.

Three review articles focus on the molecular aspects of HTLV-1 biology. Mohanty and Harhaj review how multiple signaling pathways modulated by the viral transactivator Tax protein synergize in the process of cell transformation [10]. They also discuss the regulation of Tax expression, required to escape replicative senescence and immune responses triggered by persistent and/or high levels of Tax expression. The review by Fochi et al. focuses on the NF-κB signalling pathway and on the microRNA regulatory network and outlines the properties of Tax against those of HBZ, the antisense protein of HTLV-1, which counterbalances and modulates Tax function [11]. Prochasson et al. address how Tax, as well as Rex, interfere with nonsense-mediated mRNA decay, a cell-intrinsic antiviral mechanism that induces the degradation of HTLV-1 viral RNAs [12]. This review also discusses how HTLV-1′s interference with nonsense-mediated mRNA decay may impact cellular gene expression, influencing signalling, senescence, apoptosis and immunity.

We will recall the year 2020 as the year where our lives were affected by one virus in one way or other. Araujo and Martin complement this Special Issue with an editorial on HTLV-1 infection and COVID-19, in which they stress the lack of clinical data on PLHTLV exposed to SARS-CoV-2 infection [13]. HTLV-1 causes immune dysfunction, and people suffering from ATL and HAM may have to receive immunosuppressive treatments; hence, PLHTLV may be at an increased risk of COVID-19 acquisition and suffer from more serious sequelae. This editorial provides guidance to clinicians and PLHTLV and calls for an urgent need for clinical research focusing on HTLV-1 and SARS-CoV-2 co-infection.

## References

[1] Poiesz B.J., Ruscetti F.W., Gazdar A.F., Bunn P.A., Minna J.D., Gallo R.C. (1980). Detection and isolation of type C retrovirus particles from fresh and cultured lymphocytes of a patient with cutaneous T-cell lymphoma. Proc. Natl. Acad. Sci. USA.

[2] Einsiedel L., Woodman R.J., Flynn M., Wilson K., Cassar O., Gessain A. (2016). Human T-Lymphotropic Virus type 1 infection in an Indigenous Australian population: Epidemiological insights from a hospital-based cohort study. BMC Public Health.

[3] Einsiedel L., Pham H., Wilson K., Walley R., Turpin J., Bangham C., Gessain A., Woodman R.J. (2018). Human T-Lymphotropic Virus type 1c subtype proviral loads, chronic lung disease and survival in a prospective cohort of Indigenous Australians. PLoS Negl. Trop. Dis..

[4] Gruber K. (2018). Australia tackles HTLV-1. Lancet Infect. Dis..

[5] Schwalb A., Pérez-Muto V., Cachay R., Tipismana M., Álvarez C., Mejía F., González-Lagos E., Gotuzzo E. (2020). Early-Onset HTLV-1-Associated Myelopathy/Tropical Spastic Paraparesis. Pathogens.

[6] Marcusso R.M.N., Van Weyenbergh J., de Moura J.V.L., Dahy F.E., de Moura Brasil Matos A., Haziot M.E.J., Vidal J.E., Fonseca L.A.M., Smid J., Assone T. (2020). Dichotomy in Fatal Outcomes in a Large Cohort of People Living with HTLV-1 in São Paulo, Brazil. Pathogens.

[7] Araujo A.Q.-C. (2020). Neurological Aspects of HIV-1/HTLV-1 and HIV-1/HTLV-2 Coinfection. Pathogens.

[8] Dykie A., Wijesinghe T., Rabson A.B., Madugula K., Farinas C., Wilson S., Abraham D., Jain P. (2020). Human T-cell Leukemia Virus Type 1 and Strongyloides stercoralis: Partners in Pathogenesis. Pathogens.

[9] Marino-Merlo F., Balestrieri E., Matteucci C., Mastino A., Grelli S., Macchi B. (2020). Antiretroviral Therapy in HTLV-1 Infection: An Updated Overview. Pathogens.

[10] Mohanty S., Harhaj E.W. (2020). Mechanisms of Oncogenesis by HTLV-1 Tax. Pathogens.

[11] Fochi S., Ciminale V., Trabetti E., Bertazzoni U., D’Agostino D.M., Zipeto D., Romanelli M.G. (2019). NF-κB and MicroRNA Deregulation Mediated by HTLV-1 Tax and HBZ. Pathogens.

[12] Prochasson L., Jalinot P., Mocquet V. (2020). The Complex Relationship between HTLV-1 and Nonsense-Mediated mRNA Decay (NMD). Pathogens.

[13] Araujo A., Martin F. (2020). Human T leukaemia Type 1 and COVID-19. Pathogens.

